# Extracellular Polymeric Substance Protects Some Cells in an *Escherichia coli* Biofilm from the Biomechanical Consequences of Treatment with Magainin 2

**DOI:** 10.3390/microorganisms9050976

**Published:** 2021-04-30

**Authors:** Helen M. Greer, Kanesha Overton, Megan A. Ferguson, Eileen M. Spain, Louise E. O. Darling, Megan E. Núñez, Catherine B. Volle

**Affiliations:** 1 Department of Biology, Cottey College, Nevada, MO 64772, USA; hgreer@student.cottey.edu (H.M.G.); koverton@student.cottey.edu (K.O.); 2 Department of Chemistry, State University of New York, New Paltz, NY 12561, USA; fergusom@newpaltz.edu; 3 Department of Chemistry, Occidental College, Los Angeles, CA 90041, USA; emspain@oxy.edu; 4 Department of Biological Sciences and Program in Biochemistry, Wellesley College, Wellesley, MA 02481, USA; ldarling@wellesley.edu; 5 Department of Chemistry and Program in Biochemistry, Wellesley College, Wellesley, MA 02481, USA; mnunez@wellesley.edu; 6 Departments of Biology and Chemistry, Cornell College, Mount Vernon, IA 52314, USA

**Keywords:** biofilm, magainin 2, atomic force microscopy, cell stiffness, surface roughness

## Abstract

Bacterial biofilms have long been recognized as a source of persistent infections and industrial contamination with their intransigence generally attributed to their protective layer of extracellular polymeric substances (EPS). EPS, consisting of secreted nucleic acids, proteins, and polysaccharides, make it difficult to fully eliminate biofilms by conventional chemical or physical means. Since most bacteria are capable of forming biofilms, understanding how biofilms respond to new antibiotic compounds and components of the immune system has important ramifications. Antimicrobial peptides (AMPs) are both potential novel antibiotic compounds and part of the immune response in many different organisms. Here, we use atomic force microscopy to investigate the biomechanical changes that occur in individual cells when a biofilm is exposed to the AMP magainin 2 (MAG2), which acts by permeabilizing bacterial membranes. While MAG2 is able to prevent biofilm initiation, cells in an established biofilm can withstand exposure to high concentrations of MAG2. Treated cells in the biofilm are classified into two distinct populations after treatment: one population of cells is indistinguishable from untreated cells, maintaining cellular turgor pressure and a smooth outer surface, and the second population of cells are softer than untreated cells and have a rough outer surface after treatment. Notably, the latter population is similar to planktonic cells treated with MAG2. The EPS likely reduces the local MAG2 concentration around the stiffer cells since once the EPS was enzymatically removed, all cells became softer and had rough outer surfaces. Thus, while MAG2 appears to have the same mechanism of action in biofilm cells as in planktonic ones, MAG2 cannot eradicate a biofilm unless coupled with the removal of the EPS.

## 1. Introduction

While some bacteria have a free-swimming, planktonic lifestyle, most bacteria have the ability to switch phenotypes and create complex, surface-adhered communities called biofilms [1,2]. Once they have found a suitable surface, generally in a nutrient-rich area, bacteria use a combination of pili, fimbriae, and secreted biomolecules to adhere to the surface. These bacteria then grow and divide, creating a complex, three-dimensional colony structure, complete with channels allowing the movement of water and nutrients to different parts of the biofilm. As the cells in the biofilm grow, they build a layer of extracellular polymeric substances (EPS) containing nucleic acids, various polysaccharides, and proteins, which offers both protection and stability [3]. Bacteria in the biofilm can perform specialized functions and communicate with each other using secreted autoinducer molecules [4]. When conditions no longer favor biofilm growth, individual motile cells or cell aggregates are released from the biofilm to colonize new areas [5]. Due to their protective EPS layer and ability to release cells into their environment, bacterial biofilms have long been recognized as a source of persistent infections [6]. Outside of the body, biofilms are also known sources of industrial and agricultural contamination, both as a source of pathogenic organisms and as a cause of equipment failure [7,8].

As biofilms play a significant role in disease, it is important to understand how they might react to immune defenses and/or novel therapeutics. Antimicrobial peptides (AMPs) fit into both categories. AMPs are short chains of amino acids produced as part of many eukaryotic immune responses, and they have been investigated as novel antibacterial compounds for use inside and outside the body [9]. Several classes of AMPs cause cell death by permeabilizing the bacterial membrane, which is a good target for new antibiotic treatments since developing antibiotic resistance to membrane-permeabilizing compounds would likely involve numerous mutations to the genes controlling membrane construction and maintenance [10,11,12]. The cationic magainin 2 (MAG2), originally isolated from the skin of a frog, is one such membrane-lytic AMP [13]. MAG2 is active against many different bacteria, while causing minimal damage to eukaryotic cells [13,14,15,16,17]. Given that MAG2 is positively charged, it is likely attracted to the negatively charged lipopolysaccharides (LPS) that make up the Gram-negative outer membrane and other anionic lipids that make up Gram-negative and Gram-positive cell membranes [18,19,20,21]. Initially unstructured, MAG2 can fold into an alpha helix through interactions with the bacterial membranes, allowing the MAG2 to reversibly disrupt the membrane at low concentrations [22]. As the concentration of MAG2 increases, the initial disruption by one MAG2 helix allows more MAG2 molecules to enter the membrane where they eventually form a pore, leading to unregulated release of cytosolic components.

The majority of the research investigating the ability of AMPs to kill bacteria has focused on planktonic bacteria, and as such it provides an incomplete picture of how these molecules might be used as part of the immune response or how AMPs might be used to eradicate an environmental biofilm. EPS makes removing an established biofilm difficult by providing strong adhesion to a surface and acting as a barrier to diffusion of antimicrobial molecules, potentially including AMPs [3]. While other investigations into the efficacy of AMPs on biofilms have demonstrated that various cationic, alpha-helical AMPs can reduce biofilm mass [23,24], it is unclear whether the surviving bacteria can continue to grow and restore the biofilm. Furthermore, the biomechanical effect of AMP treatment on cells in the biofilm is not well understood, and it is unclear how a cell’s location within a biofilm might affect an AMPs mechanism of action.

We previously used atomic force microscopy (AFM) to better understand the cellular biophysics of bacteria [25,26,27]. AFM is a scanning probe technique which produces high resolution images and force-distance (FD) curves, and can be used to monitor individual cells during treatment [28]. We recently used AFM to investigate planktonic *Escherichia coli* (*E. coli*) cells treated with MAG2, demonstrating significant biophysical changes occurring over 30 min of treatment that directly correspond to the known mechanism of action for MAG2 [25]. Cells treated with MAG2 had decreased turgor pressure and increased surface roughness after treatment, illustrating that MAG2 had successfully formed pores in the inner and outer membranes of the *E. coli*, and that these cells could no longer maintain membrane integrity.

In this work, we use AFM to investigate the effect of MAG2 treatment using the same strain of *E. coli* cells grown in a biofilm and shed light on the role EPS plays in long-term survival of biofilms treated with MAG2. While planktonic cells are quite susceptible to MAG2, and MAG2 prevents biofilm formation, we were unable to stop biofilm growth in an established biofilm even with high concentrations of MAG2. An investigation of the biomechanics of cells in the biofilm revealed two populations. One population had decreased cell stiffness and increased surface roughness, similar to those values obtained in planktonic cells. The other population had maintained cell stiffness and surface roughness equivalent to that of untreated biofilm cells. However, when we applied enzymes to gently digest the outer layer of EPS, all the cells in the biofilm we measured had decreased cell stiffness and increased surface roughness, and the biofilm was fully eradicated after 24 h. These results demonstrate that MAG2 likely has the same mechanism of action in the biofilm cells but is prevented by the EPS from reaching its target in a subset of cells within the biofilm. While removing the EPS from a biofilm might not be practical for in vivo use, this combination of enzymatic digestion and AMP treatment might be useful for destroying environmental biofilms.

## 2. Materials and Methods

### 2.1. Antimicrobial Peptide Preparation

The antimicrobial peptide magainin 2 (MAG2, GIGKWLHSAKKFGKAFVGEIMNS), containing a F5W mutation for easier quantitation [29], was synthesized by Genscript with >95% purity. This exchange of a phenylalanine for a tryptophan does not alter the behavior of MAG2 [29,30]. Lyophilized MAG2 was rehydrated in water to make stock solutions, and the concentration of each stock solution was determined using the absorbance at 280 nm. These stock solutions were then diluted to the appropriate concentration for further use.

### 2.2. Minimum Inhibitory Concentration of MAG2 for E. coli Biofilm Cells

The minimum inhibitory concentration for biofilm formation (MICB) was defined as the lowest concentration of MAG2 that prevented visible biofilm formation in the wells of a 48-well plate. The MICB of MAG2 was determined using a previously published protocol altered for investigating biofilm growth rather than planktonic cell growth [25,31]. After preparing *E. coli* ZK1056 (from M. O. Martin and R. Kolter) in the same manner described in [25], 100 µL of bacteria was transferred to each well in a row. MAG2 stock was diluted in Luria broth (LB) to a concentration of 264 µM and was serially diluted 1:1 across the row. The plate was incubated overnight at 37 °C, 220× rpm.

After the incubation was complete, the liquid media was observed for turbidity and then discarded. Each well was rinsed with sterile 10 mM sodium phosphate buffer at pH 7.4 (PB) before addition of a 0.1% crystal violet solution to each well. The plate was rotated to fully coat the interior surface of the wells, then incubated at room temperature for 5 min. After the incubation, the wells were rinsed with copious amounts of sterile PB. Cells in the biofilm retained the crystal violet and the wells containing a biofilm were easily identified by a purple ring at the air/water interface. The MICB determination was performed twice in triplicate.

### 2.3. Minimum Bactericidal Concentration of MAG2 on Established E. coli Biofilms

The minimum bactericidal concentration (MBC) of MAG2 was defined as the lowest concentration that killed an established biofilm, judged by the absence of turbidity in wells containing treated biofilms [32]. Two approaches were used to determine the MBC. The first was an assay similar to that used to determine the MICB. Biofilms were first grown overnight (37 °C, 220× rpm) in a 48-well plate by inoculating 75 µL of LB with 25 µL of active culture, prepared as described in [25]. The next day, the wells were observed for turbidity before the liquid media was discarded. The wells were rinsed twice with fresh LB, then 100 µL of LB was added to each well. MAG2 stock was diluted in LB to concentrations ranging from 264 µM to 0.9 mM before being serially diluted 1:1 along the row. After MAG2 addition, the plate was again incubated overnight at 37 °C, 220× rpm. After incubation, the liquid in each well was checked for bacterial growth; a well with turbid media was considered to have planktonic growth originating from the biofilm.

In the second approach, biofilms were grown as previously described for AFM experiments [26,27]. Briefly, modified microcentrifuge caps were used to hold circular glass coverslips upright in the wells of a 24-well plate. One mL of LB was added to each well and a single colony of *E. coli* was used to inoculate the culture. The plate was placed in a shaking incubator at 37 °C and 220× rpm and allowed to grow overnight. In the morning, each well was examined for turbidity in the liquid medium as well as biofilm growth on the coverslip. To better visualize the biofilm growth, some coverslips were stained with a 0.1% crystal violet solution. 

After establishing the biofilm, the glass coverslips were gently rinsed with LB and transferred to a new 24-well plate containing sterile microcentrifuge caps, fresh LB, and various concentrations of MAG2. The new plate was incubated overnight at 37 °C, 220 rpm. The next day the liquid media was inspected for turbidity, and the coverslips were removed from the wells, rinsed with PB, and observed using crystal violet, fluorescence microscopy, and atomic force microscopy (AFM).

### 2.4. Live/Dead Cell Staining

Biofilms grown on circular coverslips were stained using the Invitrogen LIVE/DEAD BacLight Bacterial Viability Kit (Carlsbad, CA, USA), following the manufacturer’s instructions. After rinsing the biofilm with PB, biofilms were treated with MAG2 or PB for 30 min. Each sample was rinsed with PB before applying 50 µL of stain to the coverslip. The samples were allowed to incubate in the dark for 15 min at room temperature before imaging with a Nikon Ti microscope using a Nikon PlanApo Lambda 40× objective (Melville, NY, USA), with a numerical aperture of 0.9. Images of cells stained with SYTO 9 were captured using a Nikon ET-GFP filter, while cells stained with propidium iodide (PI) were imaged with a Nikon ET-mCherry filter. The image resolution was 512 × 512. Images were then imported to ImageJ, where they were adjusted for brightness, overlaid, and a scale bar was added.

### 2.5. AFM

Surface images of bacteria in the biofilm were obtained in intermittent contact mode using an MFP-3D BIO AFM (Asylum Research, Santa Barbara, CA, USA), in PB as previously described [25,26,27]. Triangular silicon nitride cantilevers with a nominal spring constant of 0.32 N/m and pyramidal tips (tip height = 2.5–8.0 µm, tip radius of curvature = 10 nm) were used to obtain both images and cellular force data. In order to obtain continuous image and force data, biofilms grown on coverslips were submerged in a 50 mm Petri dish containing PB and modified for injection with a syringe. Force data, designated time zero, were acquired on the center of each cell.

After initial images and force data were obtained, a concentrated solution of MAG2 was injected into the Petri dish, allowing continuous data collection on the cells. Force data was collected every five minutes as described in [27], with five force curves acquired per location. Low resolution images were obtained in between force acquisition to ensure that cells were intact and remained in place. Final high-resolution images were taken after the final force data were obtained, and surrounding areas of the biofilm subsequently imaged.

Force data consist of both an extension and retraction curve [28]. As previously described, the extension curve is composed of three regimes: the linear approach, the nonlinear transition, and the linear compression. The slope of the linear compression (*k*_effective_, N/m) was determined using the MFP-3D software in Igor Pro and used in Equation (1) to calculate the cellular spring constant (*k*_cell_) (as in Equation (1)).

(1) $$\frac{1}{k_{\mathrm{effective}}}=\frac{1}{k_{\mathrm{cell}}}+\frac{1}{k_{\mathrm{cantilever}}}$$

Changes in the root mean squared roughness (*Rq*) of the images were determined as previously described [25].

### 2.6. Enzymatic Degradation of the Biofilm Extracellular Polymeric Substance (EPS)

Digestion of the saccharide and DNA components of the EPS by dextranase and DNaseI enzymes, respectively, was used to remove the EPS surrounding the biofilm while maintaining cell viability and adhesion to the coverslip. Twenty-five units of dextranase and 10 units of DNaseI were applied to a biofilm coated coverslip, which was then incubated at 30 °C for 30 min. After the incubation was complete, the coverslips were gently rinsed with PB to remove both the enzymes and any detached EPS.

A concanavalin A-Alexa Fluor 350 probe (Invitrogen), which selectively binds to α-mannopyranosyl and α-glucopyranosyl residues, was used to monitor EPS degradation. In order to observe both the EPS and cell viability, the live/dead stain concentrate was mixed with the concanavalin A probe concentrate and PB to create a 1X live/dead/concanavalin A solution (L/D/C). Fifty microliters of the 1× L/D/C solution was applied to either a native biofilm or a biofilm treated with dextranase and DNaseI. The biofilm was then incubated for 30 min in the dark at room temperature before being rinsed with PB and imaged as described above. The concanavalin A conjugate was imaged using a Nikon ET-DAPI filter.

## 3. Results and Discussion

### 3.1. MAG2 Prevents E. coli Biofilm Formation

We were initially interested in whether MAG2 could prevent *E. coli* ZK1056 cells from forming a biofilm. ZK1056 is a robust biofilm-forming strain and adheres strongly to surfaces, so killing the bacteria or otherwise disrupting their initial attachment to a surface would be a good strategy to prevent biofilm formation. Planktonic cells were incubated with decreasing concentrations of MAG2 and biofilm formation was assessed using crystal violet staining. The minimum inhibitory concentration for biofilm formation (MICB), the lowest concentration of MAG2 tested that prevented visible *E. coli* biofilm growth, was 16.5 µM. Interestingly, 16.5 µM was also the minimum inhibitory concentration for planktonic growth (MICP), as every well that lacked biofilm growth also lacked planktonic growth. We previously measured the MICP in an assay specific for planktonic growth, which also resulted in a MICP of 16.5 µM [25]. In experiments similar to ours, MAG2 added at the MICP was able to inhibit overnight biofilm formation by *Streptococcus mutans* [32] and drug-resistant *Acinetobacter baumannii* (*A. baumannii*) [33] strains, though the exact mechanism of biofilm prevention is unclear. As in our experiments, the MICP was equal to the MICB in both previous studies.

As the MICB and MICP are the same, it is likely that MAG2 is killing the cells before they can attach to a surface, rather than simply preventing interaction between the cells and the surface. If MAG2 was only preventing bacteria from attaching to the surface, the MICP should be higher than the MICB, as observed for other AMPs that exhibit indirect mechanisms of anti-biofilm activity. Several AMPs, such as LL-37 [34,35,36], lactoferrin [37], and IDP-1018 [38,39,40], can prevent biofilm growth at sub-MICP concentrations, indicating that these AMPs prevent or reduce attachment to a surface without killing the cells. Surface-attached magainins 1 and 2 have been shown to reduce bacterial adhesion, but the mechanism involved is unclear [41,42,43].

### 3.2. An Established E. coli Biofilm Can Withstand High Concentrations of MAG2

While MAG2 is capable of preventing biofilm formation, we also wanted to understand how MAG2 could be used to combat established biofilms. We attempted to determine the minimum bactericidal concentration (MBC) for MAG2 on mature biofilms that had grown overnight in a 48-well plate. As any planktonic growth that occurred with the initial biofilm growth was removed before the addition of MAG2, any new planktonic growth, observed as turbidity of the liquid media, must originate from cells in the biofilm. We began by using the same MAG2 concentrations used in the MICB, but we observed new planktonic growth even at the highest MAG2 concentration, 132 µM. We further increased the concentration of MAG2, eventually using 450 µM, but we observed new planktonic growth at every MAG2 concentration tested. We then switched to a coverslip-based assay, which allowed us to treat biofilms with up to 2.4 mM MAG2, and still observed new growth after overnight treatment. In either assay, MAG2 was unable to prevent continued bacterial growth at each condition tested.

### 3.3. MAG2 Permeabilizes Cells in the Biofilm

**Figure 1:**
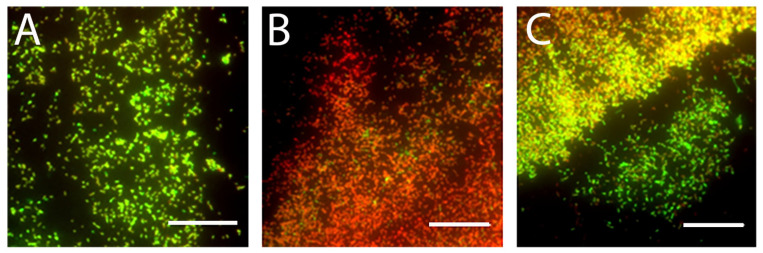
Representative images from live/dead staining of untreated and treated *E. coli* biofilms. (**A**) Biofilms grown overnight. (**B**) Biofilms treated with 16.5 µM MAG2 for 30 min. (**C**) Biofilms treated with 16.5 µM MAG2 for 24 h. The scale bar is 50 µm. Cells that appear green are stained with SYTO 9, a membrane permeable dye that enters all cells, while cells that appear red are stained with propidium iodide, a membrane impermeable dye that can only access cells once the bacterial membranes have lost integrity.

In order to better understand the interaction between MAG2 and cells in the biofilm, we performed live/dead staining on treated and untreated biofilms (Figure 1). We chose to perform experiments at the MICP so that we could compare our results using biofilm cells to those previously obtained with planktonic cells [25]. We treated biofilms grown on glass coverslips with 16.5 µM MAG2 diluted in PB for 30 min or 24 h before staining with Invitrogen’s BacLight Bacterial Viability Kit [44]. The “live” stain, SYTO 9, freely crosses bacterial membranes and fluoresces when bound to DNA, staining all cells green. The “dead” stain, propidium iodide (PI), fluoresces red when bound to DNA and exhibits a higher DNA-binding affinity than SYTO 9. However, PI can only cross the bacterial outer membrane, not the cell membrane, which prevents it from accessing the bacterial genome in healthy cells [45]. Degradation of the cell membrane allows PI to access the bacterial genome, where it outcompetes SYTO 9, and stains cells red. Notably, live/dead staining can overestimate the number of dead cells in a biofilm when PI binds to the DNA in the EPS [46], making the comparison of treated and untreated biofilms important to accurately judge the relative number of cells with permeabilized membranes. PI binding to the DNA in the EPS also explains why images of biofilms often contain overlapping regions of green and red. Cells should not retain both SYTO 9 and PI [46], but while the cells are stained green, the surrounding EPS stains red, leading to yellow regions when the images are overlaid.

Images were obtained at the edge of the biofilm, as cells in this region provide the most reliable AFM data (vide infra). Untreated biofilms have a mixture of red and green cells (Figure 1A), which is expected since some cells in the biofilm naturally die as the biofilm matures. However, the biofilm contains noticeably more red cells after a 30 min treatment with 16.5 µM MAG2 (Figure 1B), which indicates the MAG2 is capable of permeabilizing the cells in this region of the biofilm. However, the presence of green cells after 30 min of MAG2 treatment suggests that some cells escape permeabilization and could go on to regrow the biofilm. Indeed, after the biofilm is treated with MAG2 for 24 h, the biofilm is populated by both red and green cells, in a similar ratio to that observed in the untreated biofilm (Figure 1C). While it appears that MAG2 can reduce the number of viable cells in the biofilm, the remaining unaffected cells are able to quickly repopulate.

Different AMPs have varied effects on mature biofilms, and there is conflicting information on the effectiveness of individual AMPs [23]. However, most AMPs that kill cells through pore formation are less effective at eradicating established biofilms than they are at preventing new biofilm formation. MAG2 has previously shown limited effects on established *P. aeruginosa* and *A. baumannii* biofilms [33,47]. LL-37, which was able to effectively prevent biofilm formation by *S. aureus* [34], had no effect when applied to established biofilms of several clinical *S. aureus* strains, even at concentrations much higher than the MICB [48]. However, in experiments carried out by other groups, LL-37 was able to reduce the mass of *S. aureus* [49,50], *Staphylococcus epidermidis* [49], and *P. aeruginosa* [47] cells in a mature biofilm, though it is unclear if the remaining cells in the biofilm were alive or dead. Another AMP derived from frog skin, esculentin, formed pores in *P. aeruginosa* biofilm cells leading to a detectible release of β-galactosidase, but is far less active on biofilm cells than planktonic cells [51].

The inability of antibiotics to fully eradicate bacterial growth is a common problem with biofilms [52,53,54]. The presence of a layer of thick EPS can act as a physical or chemical barrier to antibiotics, preventing them from reaching their bacterial target in high enough concentrations to exert an effect. Given the presence of negatively charged extracellular DNA in the EPS, it is possible that the EPS traps some percentage of the positively charged MAG2, preventing it from reaching the bacterial membranes at the concentrations necessary to kill the cell [55]. The surviving cells could also be protected by their position within the biofilm [53]. By the time MAG2 reaches cells in the lower layers of the biofilm, it is likely present at too low a concentration to kill the cells outright. As the EPS and cells in the surface layers of the biofilm act like a sponge and sequestered MAG2, these unaffected cells can repopulate the biofilm.

### 3.4. AFM Reveals Different Cell Stiffness for Affected and Unaffected Cells

To better understand the interaction of MAG2 with individual biofilm cells, we employed atomic force microscopy (AFM) to probe the biophysical properties of biofilm cells during treatment with the antimicrobial peptide. Force distance (FD) measurements, made up of an extension and a retraction curve, can reveal important biomechanical information about cells in the biofilm [28]. In the extension curve, the tip is lowered to the bacterial surface while the resistance encountered by the tip is monitored (Figure S1 (Supplementary Materials)). When the tip first comes into contact with the surface of a cell in the biofilm, there is an initial nonlinear change in the extension curve as the tip compresses the soft layer of EPS and the outer bacterial membrane. Eventually, the tip encounters resistance generated by the bacterial turgor pressure, producing the linear region of the extension curve [26,56,57]. The slope of the linear compression represents the *k*_effective_ and is used in equation 1 to calculate the cellular spring constant *k*_cell_, which reflects the stiffness of the cell. The tip is then retracted and the FD measurement repeated before the tip is moved to a new location.

**Figure 2:**
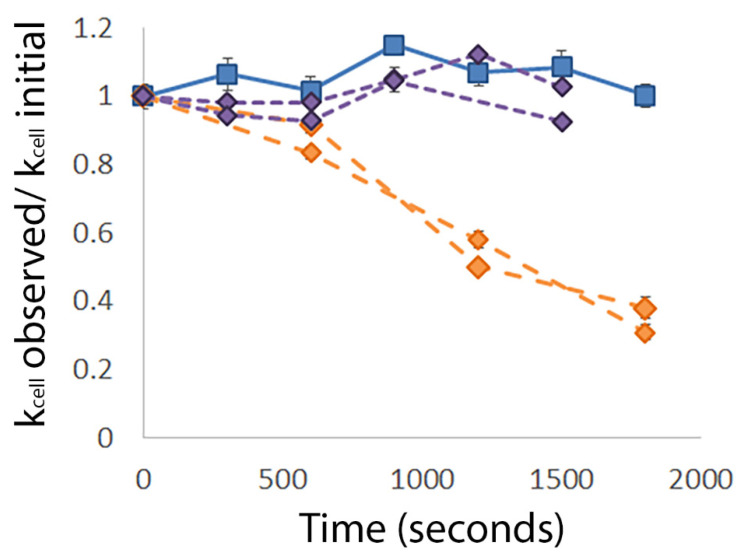
Treatment with MAG2 causes a decrease in stiffness in some biofilm cells but not in others. FD measurements were taken on cells from untreated (blue squares) and treated (purple and orange diamonds) biofilms over a 30 min time period and the *k*_cell_ was calculated as described in Equation (1). Each line represents a single cell and each point represents the average *k*_cell_ calculated from 5 FD measurements. Error bars represent the standard deviation, though the value is sometimes too small to see. To account for the heterogeneity of cell stiffness within the population, each average was normalized to the *k*_cell_ at time 0. These data are representative of the cells observed (untreated *n* = 14, treated *n* = 36).

To investigate the interaction between MAG2 and cells in the biofilm, we first analyzed untreated *E. coli* biofilms grown overnight on glass coverslips. Bacteria from the periphery of the biofilm were chosen to ensure that the measurements were from an individual cell, rather than cells piled on top of each other, which would confound the calculation of cell stiffness [26,27]. After obtaining an initial image, we collected force data every 5 min over a 30 min period. The spring constant of individual cells in the untreated biofilm fluctuated over time, indicating that maintaining turgor pressure in the biofilm is a dynamic process (Figure 2, blue squares).

To better observe the initial interaction between MAG2 and the biofilm, images and measurements were obtained before injecting MAG2 to a final concentration of 16.5 µM and collecting force data as described above. After treatment with MAG2, cells could be separated into two groups based on their stiffness (Figure 2, diamonds). The first group maintained their turgor pressure over the course of the experiment (Figure 2, purple diamonds). Though their cell stiffness did fluctuate, those fluctuations were similar to the changes observed in untreated cells. The second group was unable to maintain turgor pressure and initiated a dramatic drop in cell stiffness 5–10 min after the addition of MAG2, losing almost 75% of their stiffness after 30 min (Figure 2, orange diamonds). Planktonic *E. coli* cells treated with MAG2 at the same concentration also had decreased cell stiffness, caused by the inability of the cell to control the movement of fluids through MAG2 pores [25]. It is likely that these soft cells lost turgor pressure due to MAG2 pore-formation, while the stiff cells were able to maintain an intact membrane.

While previous studies have shown that pore-forming AMPs can decrease the cell stiffness of planktonic cells [25,58,59], few studies have measured cell stiffness in biofilm cells treated with AMPs, and we are unaware of other studies that have followed individual cells over time. Freudenthal et al. treated an *E. coli* biofilm with the linear AMP catestatin and also observed a decrease in cell stiffness, though they did not observe the two populations we describe here [60]. However, the biofilms they used were given less time to develop than those we investigated. They allowed biofilm growth to occur for only 5.5 h before addition of catestatin, which could explain the increased percentage of affected cells in the biofilm. In a similar study, Quilès et al. treated *Pseudomonas fluorescens* biofilms grown for 6 h with a derivative of the AMP dermaseptin S4 and also observed a decrease in cell stiffness, though the effect of this AMP is not as dramatic [61].

### 3.5. Soft Cells Develop a Rough Outer Surface

**Figure 3:**
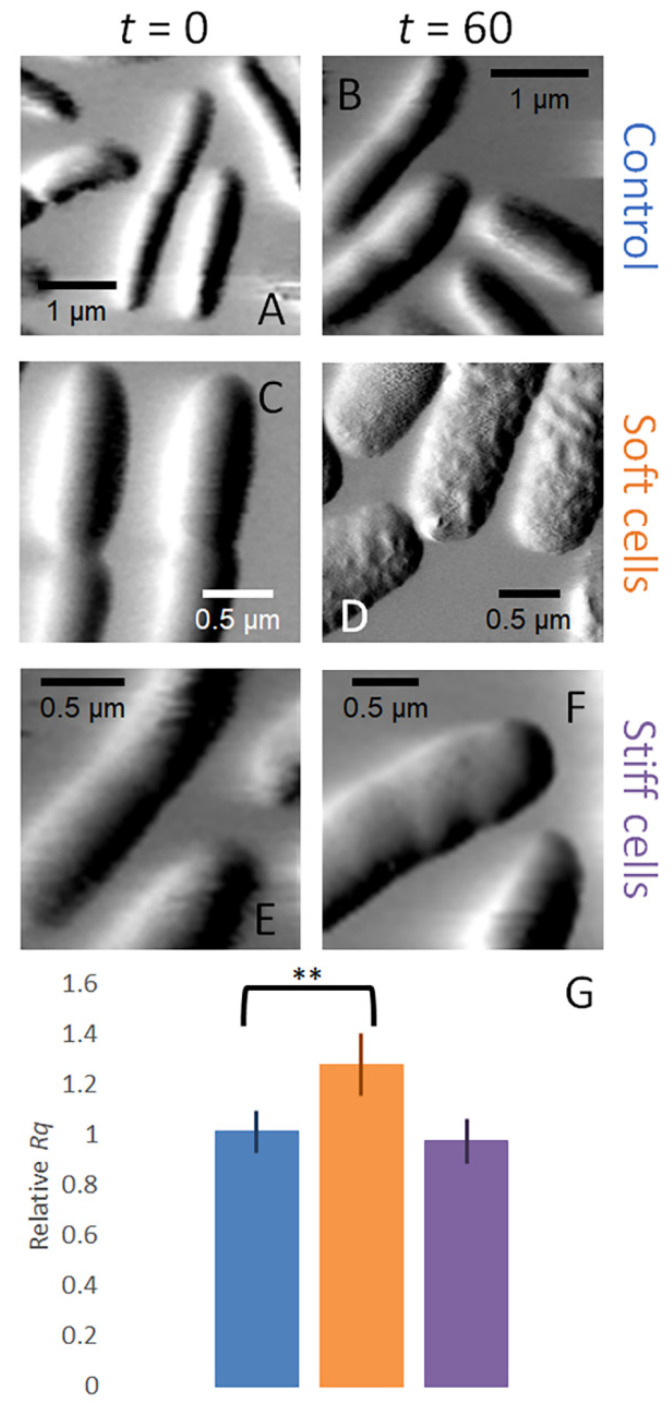
Amplitude images of *E. coli* biofilm cells captured by AFM. (**A**) Cells in an untreated biofilm at *t* = 0 min. (**B**) Cells in an untreated biofilm at *t* = 60 min. (**C**) Soft cells in a treated biofilm at *t* = 0 min. (**D**) Soft cells in a treated biofilm at *t* = 60 min. (**E**) Stiff cells in a treated biofilm at *t* = 0 min. (**F**) Stiff cells in a treated biofilm at *t* = 60 min. (**G**) The relative *Rq* for untreated cells (blue), affected soft cells (orange), and unaffected stiff cells (purple). The error bars represent standard deviation and the affected cells have a significantly higher *Rq* than the untreated cells (*p* ≤ 0.005). The data presented here are from different experiments, but are representative of the cells imaged (untreated *n* = 23, treated *n* = 57). ** *p* ≤ 0.005.

AFM is routinely used to provide high-quality images of various biological surfaces [62]. Initial images were obtained before the injection of PB (untreated) or MAG2 (treated) into the sample cell and final images were obtained after a 60 min incubation. The images of the cells in the untreated biofilm show that the cell surfaces are relatively smooth in both the initial image (Figure 3A) and in an image obtained at 60 min (Figure 3B). These images are consistent with both AFM images obtained of other biofilms [63,64,65] and previous AFM images of biofilms formed by this strain of *E. coli* [26,27].

When cells are treated with 16.5 µM MAG2, the two populations of cells with different cell stiffnesses (soft cells that had lost turgor pressure and stiff cells that have maintained turgor pressure) can also be identified by the surface morphology of the cell after treatment. Soft cells were initially indistinguishable from the untreated cells (Figure 3C), but the cell surface develops a pockmarked appearance after 60 min of treatment with 16.5 µM MAG2 (Figure 3D). The stiff cells remain indistinguishable from the untreated cells (Figure 3E,F); they both maintain their turgor pressure and retain a smooth outer surface during the 60 min incubation with MAG2. We quantified the observed changes in surface morphology by determining the root mean square roughness (*Rq*) of the surface at time 0 and at 60 min (Figure 3G). In untreated cells and stiff cells, the *Rq* values remain relatively constant. However, the soft cells have a significantly higher *Rq* after treatment. These biofilm cells strongly resemble planktonic cells treated under the same conditions [25], further supporting our conclusion that the soft cells have succumbed to MAG2 pore formation.

Most experiments where AFM is used to characterize bacteria in a biofilm either do not follow cells in the biofilm over time or are investigating changes to the gross structure of the biofilm rather than changes to the bacterial surface. However, one paper did characterize the *Rq* of *Helicobacter pylori* (*H. pylori*) biofilms before and after treatment with the macrolide antibiotic clarithromycin [66]. They did not observe any significant increase in surface roughness after treatment; since clarithromycin reduces bacterial protein production rather than targeting the bacterial membranes, clarithromycin is not expected to affect the cell surface. However, the fact that treatment with a different antibiotic did not lead to surface roughening suggests that this effect is directly related to MAG2 treatment and disruption of the bacterial membranes rather than a general feature of cell death in the biofilm.

We [25] and others [59,67] have imaged planktonic cells treated with AMPs and observed a similar roughening of the cell surface. These measurements, as with our measurements of the biofilm, were taken in buffered aqueous solutions so the appearance of a rough cell surface could not be attributed to dehydration of the cell as it was imaged. The roughened surface observed in planktonic cells and in the soft biofilm cells is consistent with pore-forming AMPs remodeling the bacterial outer surface, whereas the stiff cells likely maintain their membrane integrity. Which category a cell falls into, soft or stiff, could be related to the EPS modulating the local concentration of MAG2.

### 3.6. Removing the EPS Leads to Increased Susceptibility of Cells in the Biofilm to MAG2

**Figure 4:**
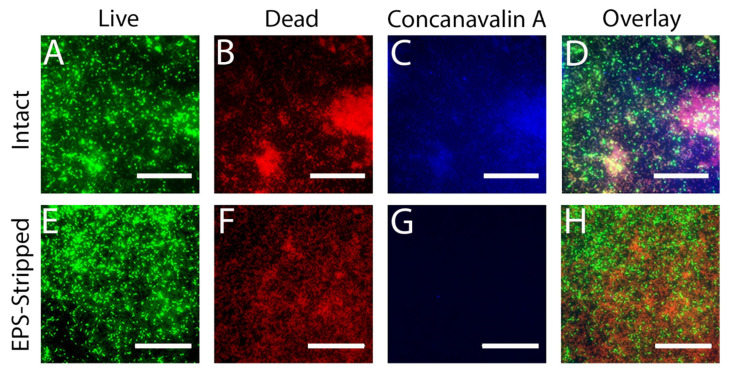
Representative images of intact (**A**–**D**) and EPS-stripped *E. coli* biofilms (**E**–**H**) stained with live/dead/concanavalin A (L/D/C) stain. (**A**) Cells stained by SYTO 9 in an intact biofilm. (**B**) Cells and eDNA stained by PI in an intact biofilm. (**C**) EPS in an intact biofilm stained by a fluorescent concanavalin A probe. (**D**) A merged image of panels (**A**–**C**). (**E**) Cells stained by SYTO 9 in an EPS-stripped biofilm. (**F**) Cells and eDNA stained by PI in an EPS-stripped biofilm. (**G**) Fluorescent concanavalin A staining in an EPS-stripped biofilm. (**H**) A merged image of panels E–G. Biofilms were grown overnight on coverslips before treatment with DNase I and dextranase to remove the EPS. After digestion was complete, the EPS-stripped biofilm and a corresponding intact biofilm were stained and imaged by fluorescent microscopy. Permeabilized bacteria have taken up the membrane impermeable PI. Bacteria with intact membranes retained the SYTO 9 dye. The fluorescently labeled concanavalin A binds to saccharides in the EPS when present. Composite images of all three channels show that the EPS layer, while robust in the intact sample, is greatly reduced in the EPS-stripped sample, but the biofilm is otherwise healthy. The scale bar is 50 µm.

While some of the cells in the biofilm are permeabilized by MAG2, others are not, and our MBC and fluorescence microscopy experiments demonstrate that the population of surviving cells continue to grow and reestablish the biofilm, even when high concentrations of MAG2 are present. We suspected that the EPS was acting as a protective barrier that the MAG2 could not fully penetrate, and that removing this defensive layer might sensitize all the cells in the biofilm to MAG2. Given that EPS is composed of DNA, protein, and various saccharides, we used DNase and dextranase to gently digest the DNA and saccharide components, removing the outer layer of EPS but leaving the cells and general biofilm structure intact [68]. We monitored the overall health of the biofilm cells using live/dead staining and used a fluorescent concanavalin A probe [69] to monitor the digestion of the EPS. Before enzymatic treatment, we observed both live (Figure 4A) and dead (Figure 4B) cells in the biofilm, along with a layer of EPS (Figure 4C) covering the biofilm surface, and the overlaid images (Figure 4D) reveal a healthy biofilm with higher concentrations of EPS in areas with high cell density. Once the biofilm was exposed to the dextranase and DNase, the EPS-stripped biofilm had a ratio of live (Figure 4E) to dead (Figure 4F) cells that is approximately the same as the intact biofilm, but there is minimal concanavalin A binding, appearing as an absence of blue signal (Figure 4G). Overlaying these images shows that even in the areas of the biofilm that have the highest cellular density, the surface layer of EPS has been successfully removed without additional harm to individual cells or major structural changes to the biofilm (Figure 4H).

**Figure 5:**
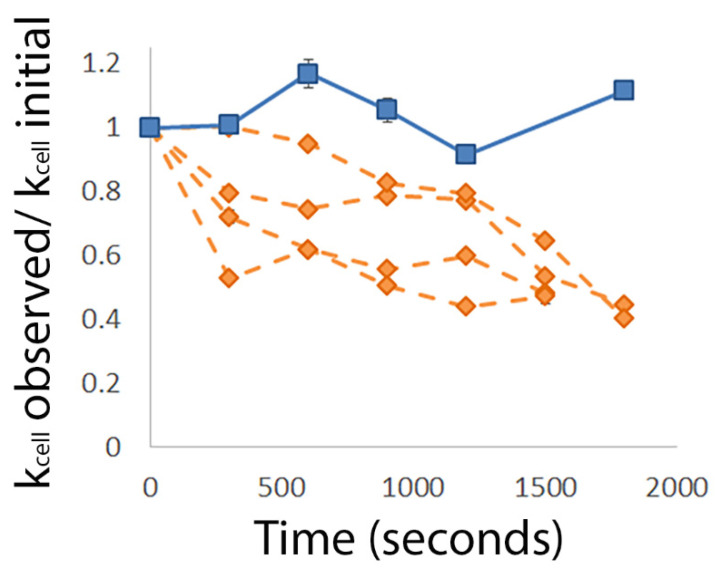
FD measurements were taken on cells in an untreated, EPS-stripped biofilm (blue squares) and on cells in an EPS-stripped biofilm treated with 16.5 µM MAG2 (orange diamonds). Each line represents measurements taken on a single cell, but is representative of the cells investigated (untreated *n* = 8, treated *n* = 16). Each point represents the average *k*_cell_ determined from 5 FD curves on a given spot in the cell. The error bars show the standard deviation, though most are too small to see.

After enzymatic digestion, the biofilm cells were examined by AFM. While the cells generally remained in place, the cells in the EPS-stripped biofilm were more fragile than those in the intact biofilm. Several cells were detached by the initial imaging process and were not included in further analysis. The cells in the untreated EPS-stripped biofilm maintained their stiffness over the course of our measurements (Figure 5, blue squares).

**Figure 6:**
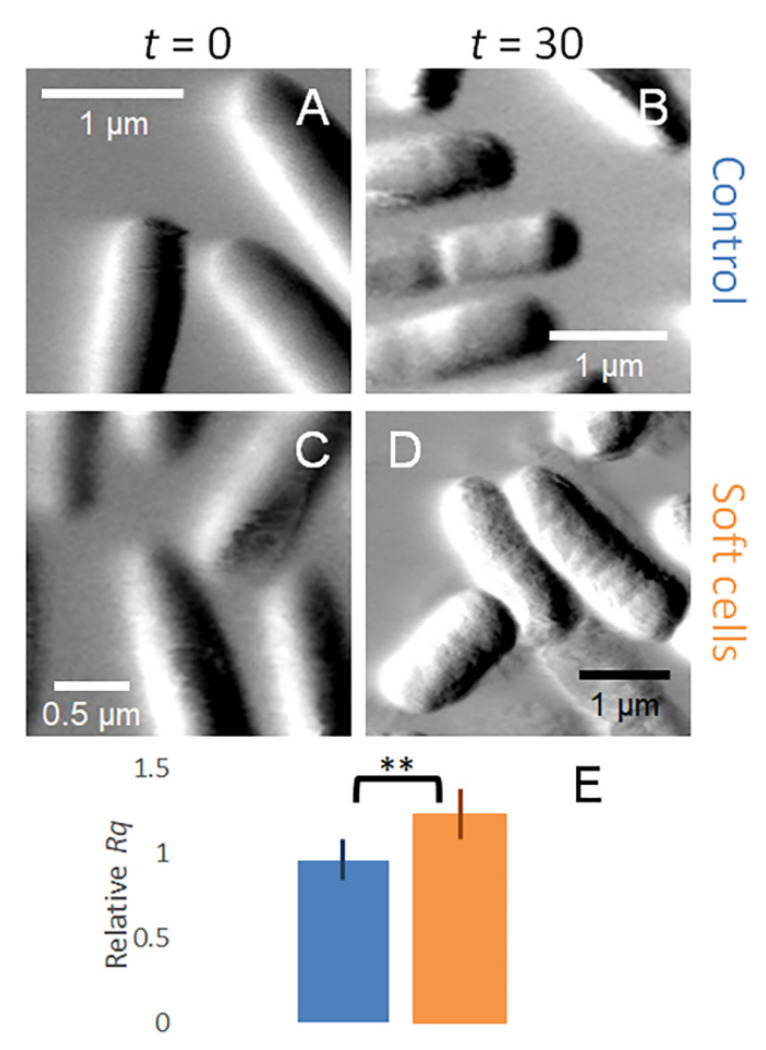
Representative amplitude images of cells in biofilms exposed to DNaseI and dextranase, which remove the EPS. (**A**) Cells in an EPS-stripped biofilm at *t* = 0 min. (**B**) Cells in an EPS-stripped biofilm at *t* = 30 min. (**C**) Cells in a MAG2-treated, EPS-stripped biofilm at *t* = 0 min. (**D**) Cells in a MAG2-treated, EPS-stripped biofilm at *t* = 30 min. (**E**) The relative *Rq* for untreated cells (blue) and MAG2-treated cells (orange). The error bars represent standard deviation and the treated cells have a significantly higher *Rq* than the untreated cells (*p* ≤ 0.005). ** *p* ≤ 0.005.

However, when we measured the stiffness of cells in the EPS-stripped biofilm treated with 16.5 µM MAG2, the stiffness of every cell we measured decreased (Figure 5, orange diamonds). Furthermore, while the surface of the untreated cells in the EPS-stripped biofilm remained relatively smooth, each MAG2-treated cell we imaged developed a roughened surface after exposure to MAG2 and the relative *Rq* for these treated cells increased (Figure 6). Overall, removing the EPS greatly increased the number of cells in the biofilm that become softer and rougher after treatment with MAG2. 

**Figure 7:**
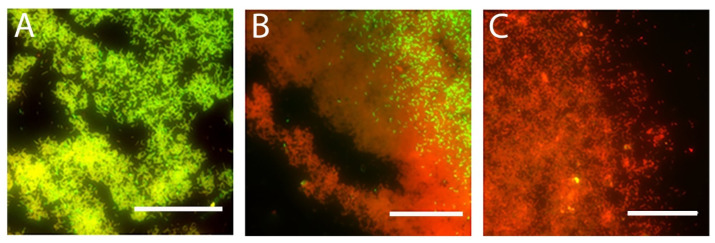
Representative images from live/dead staining of EPS-stripped biofilms. (**A**) Untreated EPS-stripped biofilms. (**B**) EPS-stripped biofilms treated with 16.5 μM MAG2 for 30 min. (**C**) EPS-stripped biofilms treated with 16.5 μM MAG2 for 24 h. Even with its EPS removed, the cells of the untreated stripped biofilm have mostly retained the SYTO 9 dye, while the cells in the treated biofilms have been permeabilized and taken up PI. Note the contrast between panel C and the biofilm with intact EPS in Figure 1C. The scale bar is 50 μm.

We also repeated the fluorescence microscopy experiments with the EPS-stripped biofilm, now focusing on the periphery of the biofilm where we obtained the AFM data. Live/dead images of the untreated EPS-stripped biofilm show a relatively healthy biofilm, with many live cells at the periphery (Figure 7A). After 30 min of MAG2 treatment, there are still some live cells, but the vast majority of cells had been permeabilized (Figure 7B). Importantly, when the EPS-stripped biofilm was incubated with 16.5 µM MAG2 for 24 h, all the cells in the biofilm took up PI, indicating that all the cells in the biofilm were permeabilized (Figure 7C). Furthermore, when the EPS-stripped biofilm was incubated overnight with MAG2, there was no accompanying planktonic growth, indicating that combining enzymatic digestion of the DNA and saccharide components and the application of MAG2 eradicated the biofilm (Figure S2 (Supplementary Materials)).

Other studies have used various enzymes, particularly proteases, to enhance the effectiveness of conventional antimicrobial compounds on biofilms. Using 3-day-old *S. aureus* and *S. epidermidis* biofilms, Baidamshina et al. showed that ficin, a nonspecific protease derived from plants, could reduce biofilm mass, and observed a further reduction in mass when ficin was used in conjunction with ciprofloxacin [65]. By removing the alginate from *H. pylori* biofilms, Bugli et al. also reported further decreases in biofilm mass after treatment with clarithromycin [66]. Degrading the EPS also increases the efficacy of some cleaning compounds. A mix of several different enzymes that target the major biomolecules in the EPS was able to better remove *P. aeruginosa* and *S. aureus* biofilms from endoscope surfaces than the cleaners alone [70]. However, it is unclear whether the cells remaining on the surface could repopulate the biofilm.

While most AMPs can only reduce the growth of an established biofilm, piscidin 3 (P3), an AMP first isolated from hybrid striped seabass, shows potent antibiofilm activity compared to the homologous protein piscidin 1 (P1) [71,72]. Both P1 and P3 contain an amino terminal Cu(II)- and Ni(II)-binding (ATCUN) motif and can act as a nuclease, cleaving the DNA through the production of hydroxyl radicals [73]. Libardo et al. showed that P3 is able to cleave DNA better than P1 and proposed that by first efficiently degrading the extracellular DNA in the EPS, P3 gets greater access to cells in the biofilm, leading to more cell death [71]. Here, we also used both nuclease activity (from DNaseI) and antimicrobial activity (from MAG2) to destroy an *E. coli* biofilm, further demonstrating that the combination of these two functions may have important implications for in vivo and in vitro biofilm eradication.

## 4. Conclusions

Biofilms are ubiquitous in the environment, and controlling biofilm growth is a pressing challenge. AMPs like MAG2 are part of many immune systems and have been suggested as potential novel antibiotics. Thus, understanding the interaction between biofilms and MAG2 provides important information not only on the effectiveness of these molecules as part of the immune response to biofilm formation, but also on possible applications as an antibiotic. In this work, we evaluated the efficacy of MAG2 on biofilm cells. While MAG2 is able to efficiently prevent biofilm formation, it was unable to destroy a mature biofilm at any concentration tested. Using AFM to monitor individual cells in a biofilm treated with MAG2, we observed two populations of cells. Some cells in the biofilm become softer and rougher, features we have also observed in planktonic cells and likely caused by MAG2 remodeling the outer bacterial membrane. Other cells were indistinguishable from untreated biofilm cells, maintaining their stiffness and smooth surface. Removing the outer layer of EPS caused all cells to become softer and rougher after MAG2 treatment, indicating that MAG2 likely uses the same mechanism to attack biofilm cells as it does planktonic cells, but is prevented from reaching a critical concentration at the outer membrane by the EPS. Importantly, after EPS-removal and treatment with MAG2, the biofilm was completely eradicated after 24 h. While removing the EPS may not be an ideal strategy to treat biofilms in a clinical setting, it would be a very effective strategy to sensitize environmental biofilms to AMP attack. Given the heterogeneity of environmental biofilms, further research will be necessary to determine the optimal combination of EPS degrading enzymes and AMPs, but the experiments presented here are a necessary first step.

## Supplementary Materials

The following are available online at https://www.mdpi.com/article/10.3390/microorganisms9050976/s1, Figure S1: Representative force distance curves from untreated and treated biofilms. Figure S2: Comparison of planktonic and biofilm growth from intact and EPS-stripped biofilms treated with MAG2.
