# Supplementary material for: Extracellular Polymeric Substance Protects Some Cells in an Escherichia coli Biofilm from the Biomechanical Consequences of Treatment with Magainin 2
Source: Microorganisms. 2021 Apr 30;9(5):976. doi: 10.3390/microorganisms9050976 (PMC8147140; doi:10.3390/microorganisms9050976)
Supplement: Supplementary file 1 [file microorganisms-09-00976-s001.zip › microorganisms-1194961-supplementary.pdf]

Supplemental Materials

Extracellular Polymeric Substance Protects Some Cells in an *E. coli* Biofilm from the Biomechanical Consequences of Treatment with Magainin 2

Helen M. Greer[1+], Kanesha Overton[1+], Megan A. Ferguson[2], Eileen M. Spain[3], Louise E. O. Darling[4], Megan E. Núñez[5], and Catherine B. Volle[6*]

1 Department of Biology, Cottey College, Nevada, MO 64772, USA
2 Department of Chemistry, State University of New York, New Paltz, NY 12561, USA
3 Department of Chemistry, Occidental College, Los Angeles, CA 90041, USA
4 Department of Biological Sciences and Program in Biochemistry, Wellesley College, Wellesley, MA 02481
5 Department of Chemistry and Program in Biochemistry, Wellesley College, Wellesley, MA 02481, USA
6 Departments of Biology and Chemistry, Cornell College, Mount Vernon, IA 52314, USA
+ These authors contributed equally to this work.
* Correspondence: cvolle@cornellcollege.edu; Tel.: (319) 895-4413

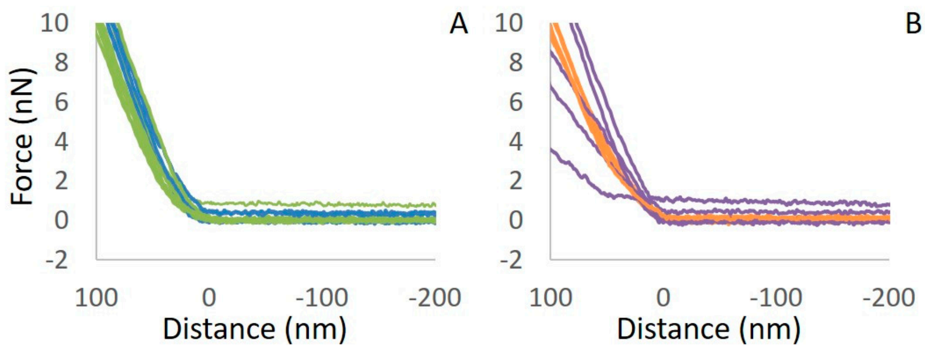

Figure S1: Representative force distance curves from untreated and treated biofilms. (A) Green curves were taken on cells at time = 0 min, while blue curves were taken after 30 min. (B) Orange curves were taken before treatment, while purple curves were taken after 30 minutes of treatment.

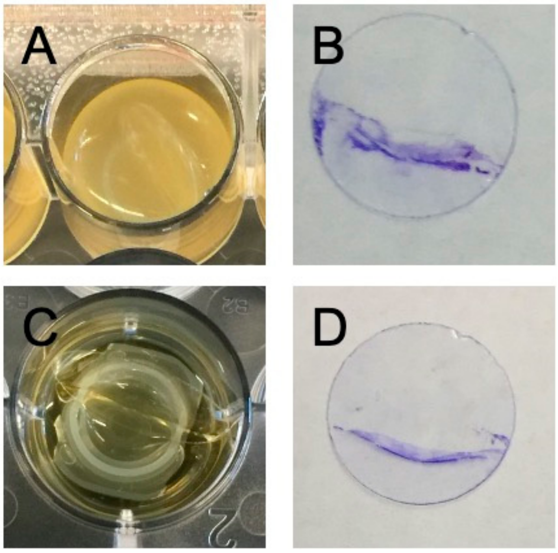

Figure S2: Comparison of planktonic and biofilm growth from intact and EPS-stripped biofilms treated with MAG2. (A) Planktonic cells grew from intact biofilms placed in wells with fresh LB and 16.5 μM MAG2, and (B) the biofilm also continued grow duing the same period. (C) When EPS-stripped biofilms were placed in a well with fresh LB and 16.5 μM MAG2, no planktonic cells grew, and (D) the biofilm did not increase in size.
